# A study of pre- and post-treatment hematologic markers of immune response in patients undergoing radiotherapy for soft tissue sarcoma

**DOI:** 10.3389/fonc.2024.1392705

**Published:** 2024-10-03

**Authors:** Eric Ku, Garrett Harada, Grace Lee, Akul Munjal, Nicholas Peterson, Jino Park, Warren Chow, Russell Stitzlein, Charles Limoli, Jeremy Harris

**Affiliations:** ^1^ Department of Radiation Oncology, University of California Irvine, Orange, CA, United States; ^2^ School of Medicine, University of California Irvine, Irvine, CA, United States; ^3^ Department of Hematology/Oncology, University of California Irvine, Orange, CA, United States; ^4^ Department of Orthopedic Surgery, University of California Irvine, Orange, CA, United States

**Keywords:** sarcoma, radiation, NLR, PLR, dosimetry

## Abstract

**Introduction:**

This study investigates the impact of pre- and post-treatment hematologic markers, specifically neutrophil-to-lymphocyte ratio (NLR) and platelet-to-lymphocyte ratio (PLR), on treatment outcomes in soft tissue sarcoma (STS) patients undergoing radiation therapy (RT).

**Methods:**

Data from 64 patients who underwent RT for curative management of STS were reviewed. Pre-RT and post-RT hematologic measures were evaluated for associations with survival outcomes. A normal tissue complication probability (NTCP) curve for predicting ΔPLR ≥ 75 was modeled using a probit function.

**Results:**

Elevated baseline NLR was associated with worse overall survival (OS) and disease-free survival (DFS), while elevated PLR was associated with worse DFS. Post-RT, elevated PLR was linked to worse OS and DFS. Increasing PLR change post-RT was associated with worse OS and DFS. Receiver operating characteristics analysis determined ΔPLR ≥ 75 to be a robust cutoff associated with worse DFS. Bone V10Gy ≥362 cc corresponded to a 50% risk of developing ΔPLR ≥ 75.

**Discussion:**

These results suggest that hematologic markers could serve as prognostic biomarkers in both pre- and post-treatment settings for STS patients undergoing RT. Future studies can consider using bone V10Gy < 362 cc as a potential cutoff to reduce the risk of increased PLR after RT.

## Introduction

Soft-tissue sarcomas (STS) constitute a heterogenous disease characterized by varied anatomical presentations and over 50 histological subtypes with disease courses spanning a wide spectrum (1, 2). Consequently, there is a necessity for improved understanding of the prognosis of STS beyond established staging criteria such as anatomical presentation, tumor size, and grading (3).

Recent studies have demonstrated that inflammatory markers obtained from routine blood tests can serve as valuable prognostic markers in different types of cancers, including STS (4–6). The presence of tumor-associated neutrophils is believed to have a critical role in promoting tumor growth and metastasis, and elevated neutrophil-to-lymphocyte ratio (NLR) has been linked to a worse prognosis in various cancer types (7–9). Similarly, platelets, which serve as acute phase reactants, have shown utility in determining cancer prognosis, with elevated platelet-to-lymphocyte ratio being associated with worse outcomes (10–12).

The management of non-metastatic STS may involve a combination of surgery and radiation (RT). Previous research has shown that pre-operative elevations in NLR and PLR are associated with worse overall survival (OS) in STS (13–16). While neutrophil, platelet, and lymphocyte progenitor cells are well-known to be sensitive to radiation, the relative impact of RT on NLR or PLR is not well understood (13–15).

Sarcomas are generally thought to have low immunogenicity and low tumor mutational burden (17, 18). However, recent clinical trials have shown promise with the use of immune checkpoint inhibitors (ICIs) in certain immunogenic subtypes such as undifferentiated pleomorphic sarcoma and de-differentiated liposarcoma (19, 20). Understanding the effects of RT on the immune response in sarcoma is crucial, especially given recent efforts to combine RT with ICIs to enhance the immune response and improve the efficacy of these therapies (21).

This study had two main objectives. First, to investigate the association between the relatively unexplored hematologic markers of immune response (neutrophil-to-lymphocyte ratio (NLR), and platelet-to-lymphocyte ratio (PLR)) with treatment and survival outcomes in patients undergoing RT. Second, to assess the impact of RT and dosimetric parameters on these hematologic markers before and after treatment.

## Materials and methods

### Patient selection

A retrospective study was conducted on patients who underwent management of non-metastatic STS at a single academic institution between September 2009 and January 2023. A total of 64 patients were included in the analysis. Treatment consisted of neoadjuvant or adjuvant radiation RT with surgery, or definitive RT alone. Chemotherapy was sometimes employed for high-risk patients or with rhabdomyosarcoma histology. Staging was performed based on criteria outlined in the 8^th^ edition of the American Joint Committee on Cancer staging.

### Hematologic assessments

Routine follow-up, at provider discretion, consisted of a physical exam, CBC with differential, and radiologic assessments. ANC, ALC, and PLT were recorded prior to the initiation of any treatment (pre-RT) and between 0 to 4 months post-RT (post-RT). To minimize the capture of transient changes resulting from infection or medication adverse effects, CBC measures with significant leukopenia (WBC < 4 x 10^3^ cells/μL) or leukocytosis (WBC > 12 x 10^3^ cells/μL) were excluded from the analysis. NLR and PLR were calculated by dividing ANC by ALC and PLT by ALC, respectively. Delta (Δ) values were calculated by subtracting the post-RT measures from the pre-RT measures.

### Dosimetric analysis

Evaluation of RT treatment plans was performed on MIM (MIM Software Inc., Cleveland, OH). Organ volumes were individually delineated for the body and bones to determine dose-volume histograms (DVH). Total dose was converted to equivalent dose in 2 Gy fractions (EQD_2_) to account for different dose regimens. Mean doses to body and bone as well as volumetric doses, defined as volume of body or bone receiving 10 Gy, 20 Gy, or 30 Gy (V10Gy, V20 Gy, V30 Gy), were recorded.

### Statistical analysis

Kaplan-Meier curves were used to estimate OS and DFS and log-rank testing was used to compare groups. OS was calculated as the duration in months from the initiation of RT to death from any cause. Disease-free survival DFS was calculated as the duration in months from the initiation of RT to disease recurrence, distant or local progression, or death. Baseline and follow-up ALC, ANC, PLT, NLR, and PLR were compared using paired T-test and effect size was estimated with Cohen’s d. Univariate and multivariate Cox proportional hazards regression models were used to analyze associations between clinical factors and hematologic markers with survival outcomes. Spearman’s rank correlation coefficient was used to evaluate for correlations between ΔPLR and dosimetric parameters. Normal Tissue Complication Probability (NTCP) for developing ΔPLR ≥ 75 was modeled with a probit regression function (22, 23). Data analysis was performed using SPSS Version 24.0 (IBM Corp., Armonk, NY) and R Version 4 (R Foundation for Statistical Computing, Vienna, Austria).

## Results

### Clinical characteristics

64 patients were included in this study (**Table 1**). Median age was 59 years old (range: 22 - 89 years old). Primary disease sites were extremities in 37 patients (58%), trunk in 12 patients (19%), and head and neck in 15 patients (23%). T1 disease was present in 9 patients (14%), T2 in 27 patients (42%), T3 in 15 patients (23%), and T4 in 13 patients (20%). N1 disease was present in 3 patients (4.7%). Grade 1 disease was found in 8 patients (14%), grade 2 disease in 6 patients (9.4%), and grade 3 disease in 50 patients (78%). Goal of radiation was definitive in 7 patients (11%) and either neoadjuvant or adjuvant in 57 patients (89%). 3D conformal technique was utilized in 17 patients (27%) and IMRT in 47 patients (73%). Median RT dose was 51 Gy (range: 39 - 78 Gy). 30 patients (47%) received chemotherapy and 54 patients (84%) underwent surgical resection as a part of their definitive management.

**Table 1:** Patient clinical characteristics.

Characteristic	
Age, median (range), yr	59 [22-89]
Follow-up, median (range), mo	23 [3-142]
Sex, No. (%)	
Male	40 (62%)
Female	24 (38%)
Location, No. (%)	
Head/Neck	15 (23%)
Upper/Lower Extremity	37 (58%)
Trunk	12 (19%)
Tumor Staging, No. (%)	
T1	9 (14%)
T2	27 (42%)
T3	15 (23%)
T4	13 (20%)
Nodal Staging, No. (%)	
N0	61 (95%)
N1	3 (4.7%)
Grade, No. (%)	
Grade 1	8 (14%)
Grade 2	6 (9.4%)
Grade 3	50 (78%)
Histology, No. (%)	
Angiosarcoma	6 (9%)
Carcinosarcoma	1 (1.5%)
Clear Cell Sarcoma	1 (1.5%)
Leiomyosarcoma	7 (11%)
Liposarcoma	18 (28%)
Dedifferentiated Liposarcoma	2 (3.1%)
Myxoid Liposarcoma	11 (17%)
Pleomorphic Liposarcoma	4 (6.3%)
Well-Differentiated Liposarcoma	1 (1.5%)
Malignant Peripheral Nerve Sheath Tumor	3 (4.7%)
Myxofibrosarcoma	1 (1.5%)
Pleomorphic Dermal Sarcoma	3 (4.7%)
Rhabdomyosarcoma	4 (6.3%)
Spindle Cell Sarcoma	3 (4.7%)
Synovial Sarcoma	4 (6.3%)
Undifferentiated Pleomorphic Sarcoma	11 (17%)
Unknown Histology	2 (3.1%)
Chemotherapy, No (%)	30 (47%)
Surgery, No (%)	54 (84%)
Radiation Technique	
3DC, No (%)	17 (27%)
IMRT, No (%)	47 (73%)
Total RT dose, median [range], Gy	51 [39-78]

### Hematologic effects

**Figure 1:**
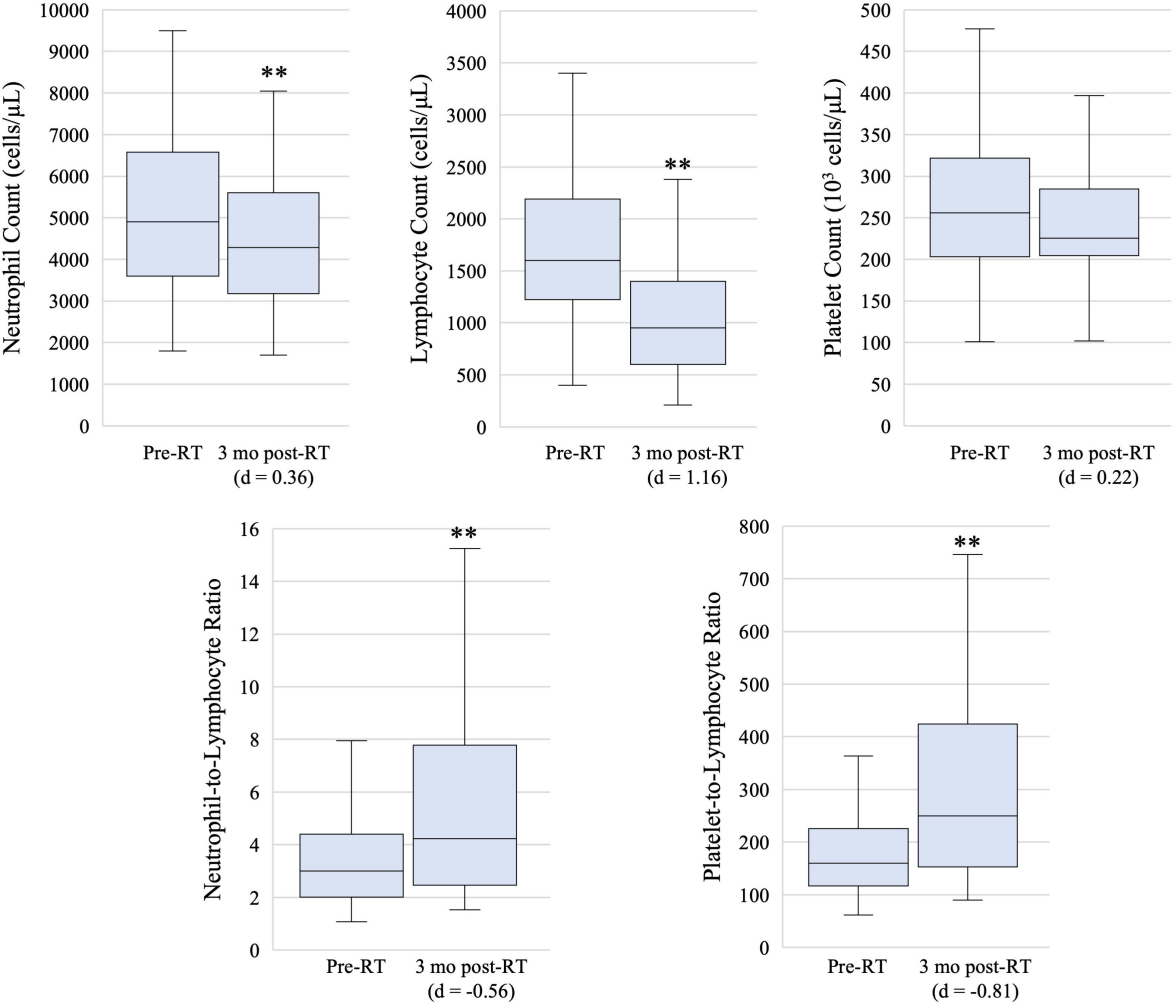
Box and whisker plots showing pre-radiation therapy (pre-RT) and 3 month post-radiation therapy (3 mo post-RT) hematologic marker measures. Box, line, and whiskers represent interquartile range, median, and 95% confidence interval, respectively. ** indicates p-value < 0.05 on paired T-test and effect size is estimated with Cohen’s d.

The median pre-RT values for ANC, ALC, PLT, NLR, and PLR were 4900 cells/μL (IQR: 3600 - 6600 cells/μL), 1600 cells/μL (IQR: 1200 - 2200 cells/μL), 256 x 10^3^ cells/μL (IQR: 203 x 10^3^ - 321 x 10^3^ cells/μL), 3.0 (IQR: 2.0 - 4.4), and 160 (120 – 230), respectively (**Figure 1**). At the 3 month post-RT time point, the median values for ANC, ALC, PLT, NLR, and PLR were 4300 cells/μL (IQR: 3200 - 5600 cells/μL), 950 cells/μL (IQR: 600 - 1400 cells/μL), 225 x 10^3^ cells/μL (IQR: 204 x 10^3^ - 284 x 10^3^ cells/μL), 4.2 (IQR: 2.5 - 7.8), and 250 (150 – 420), respectively. Significant decreases in ANC and ALC, as well as significant increases in NLR and PLR, were noted between the 3 month post-RT and pre-RT timepoints. The decrease in ALC and in the increase in PLR showed stronger effect sizes, with Cohen’s d > 0.8.

### Survival outcomes

The median follow-up duration was 23 months. The median OS was 84 months (95% CI: 57 - 112 months), and the median DFS was 20 months (95% CI: 1 - 72 months). Several baseline clinical factors were associated with worse OS on univariate Cox proportional hazards regression, including advanced T-stage disease, head and neck primary disease, receipt of chemotherapy, lack of surgical resection, receipt of definitive RT alone, and use of intensity-modulated radiation therapy (IMRT) technique (**Supplementary Table S1**). Male sex, advanced T-stage disease, receipt of chemotherapy, lack of surgical resection, and use of IMRT technique were associated with worse DFS.

**Figure 2:**
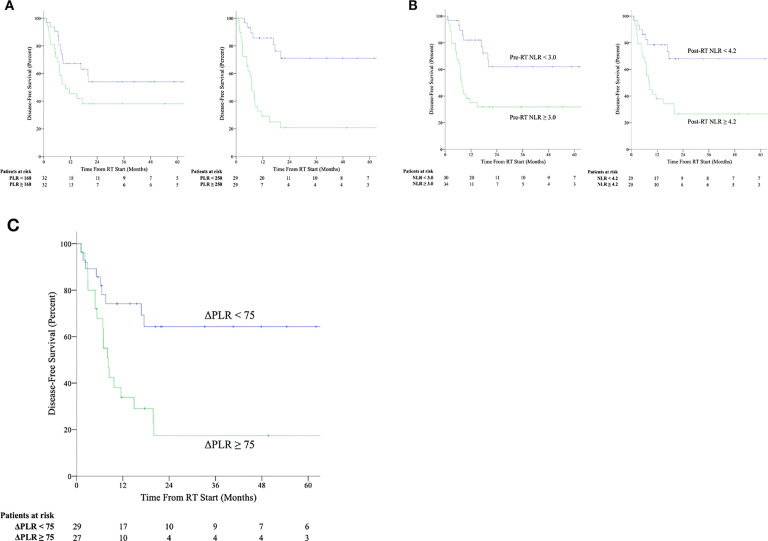
Kaplan-Meier survival curves for disease-free survival stratified by above median and below median values of platelet-to-lymphocyte ratio (NLR) **(A)** and neutrophil-to-lymphocyte ratio (PLR) **(B)** at the pre-radiation therapy (pre-RT) and post-radiation therapy (post-RT) timepoints as well as by values of change in platelet-to-lymphocyte ratio between post-radiation therapy and pre-radiation therapy timepoints (ΔPLR) ≥ 75 or < 75 **(C)**.

At the pre-RT timepoint, elevated ANC, elevated NLR, and elevated PLR were associated with worse OS (**Supplementary Table S1**). However, on multivariate regression analysis considering surgical resection and chemotherapy, only NLR remained associated with OS. Similarly, elevated ANC, elevated NLR, and elevated PLR were associated with worse DFS. All three markers remained associated with DFS on multivariate regression analysis. Kaplan-Meier curves for DFS shown in **Figures 2A, B**. DFS data by histological subtype and stratified by ΔPLR and pre-RT/post-RT NLR cut-offs is shown in **Supplementary Table S2**.

At the 3-month post-RT timepoint, lower ALC, elevated PLT, and elevated PLR were associated with worse OS. PLT and PLR remained associated with OS on multivariate regression. Lower ALC, elevated PLT, elevated NLR, and elevated PLR were associated with worse DFS. ALC, PLT, and PLR continued to show associations with DFS on multivariate regression.

ΔANC, ΔALC, ΔPLT, and ΔNLR were not associated with worse outcomes, whereas an increased ΔPLR was associated with both OS and DFS on univariate and multivariate regression (**Figure 2C**). ROC analysis was used to determine optimum cutoff values for predicting DFS (AUC 0.745, p = 0.002) and determined to be ΔPLR ≥ 75 (Sn = 66%, Sp = 75%) using the concordance probability method (24). A ΔPLR ≥ 75 was associated with a 5-year OS of 40% and 5-year DFS of 18%, compared to 74% OS (p = 0.046) and 64% DFS (p = 0.005) for those with a ΔPLR < 75.

### Dosimetric analysis

Dosimetric parameters that significantly correlated with ΔPLR were mean body dose (r_s_ = 0.29), body V10 Gy (r_s_ = 0.34), body V20 Gy (r_s_ = 0.31), body V30 Gy (r_s_ = 0.28), mean bone dose (r_s_ = 0.37), bone V10 Gy (r_s_ = 0.48), bone V20 Gy (r_s_ = 0.46), and bone V30 Gy (r_s_ = 0.38).

**Figure 3:**
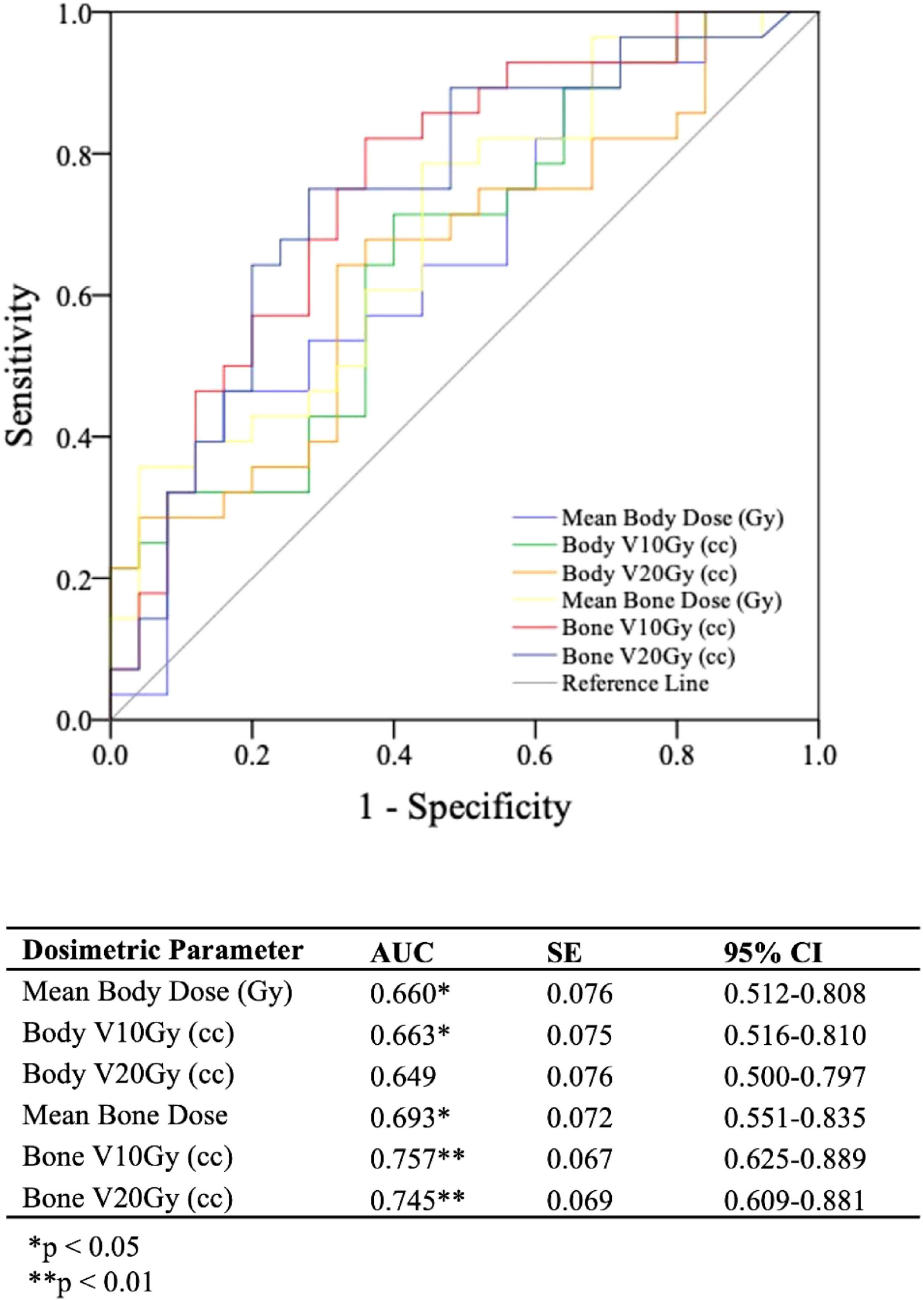
Receiver operating characteristics (ROC) curves and analysis for evaluating bone and body dosimetric parameters ability to predict a change in platelet-to-lymphocyte ratio between post-radiation therapy and pre-radiation therapy timepoints (ΔPLR) ≥ 75.

**Figure 4:**
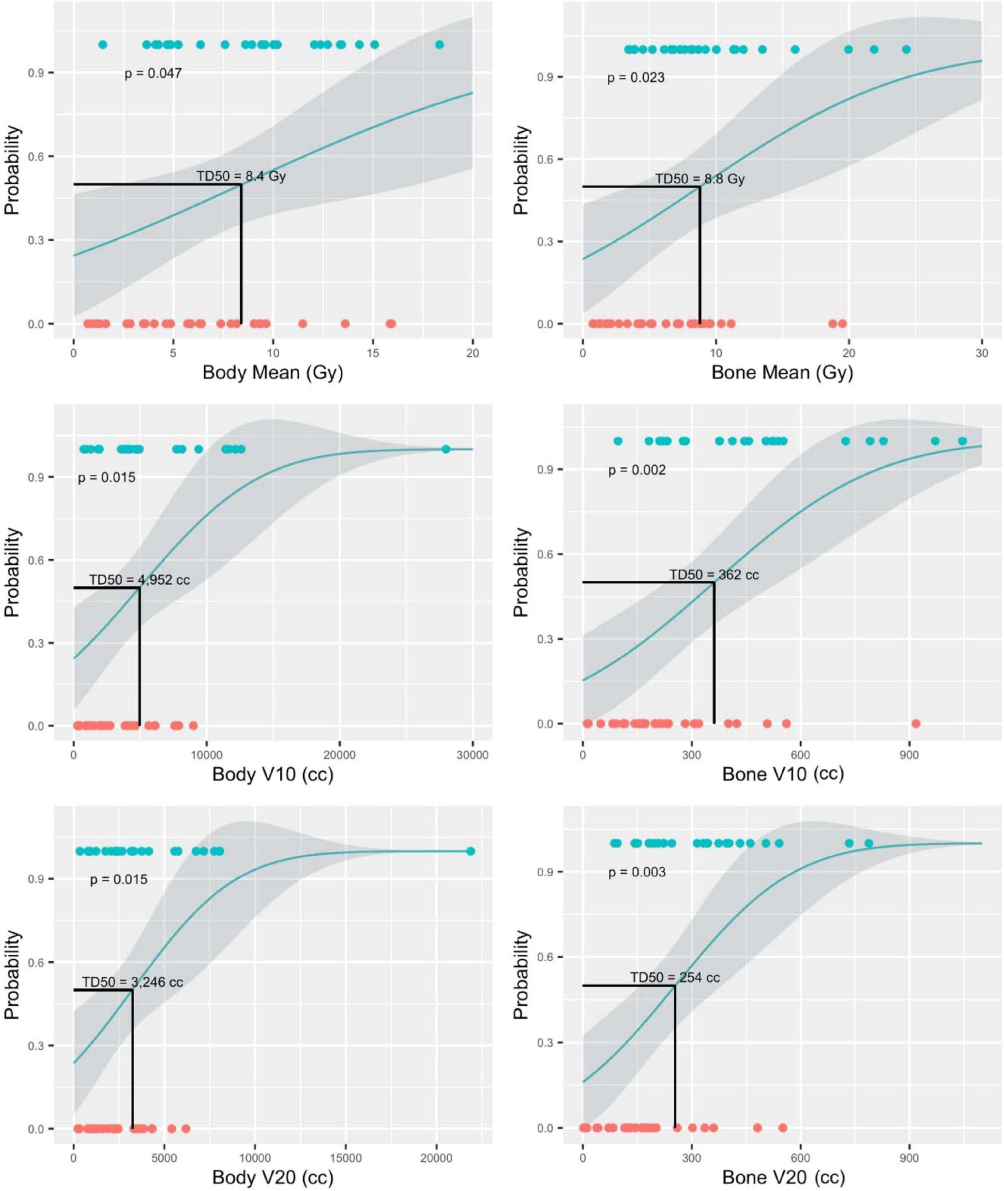
Normal tissue complication probability (NTCP) models for predicting change in platelet-to-lymphocyte ratio (ΔPLR) ≥ 75. Blue circles represent patients with ΔPLR ≥ 75 and orange circles represent patients with ΔPLR < 75.

With a ΔPLR “toxicity” of ≥ 75, dosimetric parameters and their associations with this cutoff were evaluated. Among the dosimetric parameters, bone V10Gy demonstrated the highest capability for predicting ΔPLR ≥ 75 (AUC > 0.8, p < 0.001) (**Figure 3**). The dosimetric parameter values corresponding to a 50% risk (TD50) of developing ΔPLR ≥ 75 were body mean, body V10 Gy, body V20 Gy, bone mean, bone V10 Gy, and bone V20 Gy of 8.4 Gy, 4952 cc, 3,246 cc, 8.8 Gy, 362 cc, and 254 cc, respectively (**Figure 4**).

## Discussion

Our findings were that elevated baseline NLR and PLR were associated with worse survival outcomes in patients undergoing RT for STS. Additionally, we observed a greater increase in PLR between pre-RT and post-RT measurements was associated with worse survival outcomes. We then identified a suggested threshold for bone V10Gy that could help mitigate the risk of increased ΔPLR.

There have been a number of retrospective studies that have shown the usefulness of pre-treatment NLR and PLR in determining prognosis in STS (13, 14, 16, 25–27). However, the existing evidence is conflicting, as a recent multicenter sarcoma database study found both NLR and PLR to be poor predictors of mortality and recurrence-free survival (28). Notably, the patients in that study consisted exclusively of STS of the retroperitoneum and trunk, while our study included a majority of patients with extremity tumors. Moreover, our study is unique as it focused on a subset of patients receiving RT for STS, and the results regarding prognostic utility of NLR and PLR are consistent with previous work on radiation treatment outcomes in other malignancies, including lung, esophageal, cervical, and pancreatic cancers (29–34).

The increase in NLR and PLR shortly after completing RT aligns with the sensitivity of lymphocytes to radiation and the relative lower sensitivity of neutrophil and platelet progenitor cells. However, as expected, drops were observed in all cell lines (35). Recent research has focused on radiation-induced lymphopenia and its impact on treatment outcomes across cancer subtypes (36). The capability of neutrophils to hinder lymphocyte infiltration within the tumor microenvironment plays a critical role in the tumor immune response. This is achieved in part, by inhibiting antitumor responses and releasing anti-inflammatory cytokines (37, 38). Therefore, NLR may be viewed as a balance between host pro-inflammatory and anti-inflammatory mediators. While evidence exists linking platelets to cancer progression through mechanisms like increased angiogenesis and subsequent increased metastatic risk, the precise mechanism by which elevated PLR leads to worse outcomes is not as firmly established (39–41). Another theory suggests that PLR might reflect a more intense tumor-induced host systemic inflammatory response, potentially contributing to worse outcomes (42). Notably, in our study, only an increase in ΔPLR was associated with worse overall survival. These findings underscore the need for further investigation into how radiation treatment may affect the tumor immune response, the tumor microenvironment, and certain hematologic markers such as PLR.

Several studies have examined dosimetric parameters and their influence on NLR and PLR (30, 32, 33, 43, 44). For example, Wolf et al. found NLR to be associated with worse outcomes and that increased spleen dose may contribute to a greater change in NLR after RT for locally advanced pancreatic cancer (32). However, we did not find an association between ΔNLR, ΔANC, or ΔALC and any survival outcomes, suggesting possible differences in hematologic adverse effects between treating STS and intraabdominal cancers. Another study focusing on lung cancer and chemoradiation discovered that volumetric heart doses and mean body dose were related to post-RT NLR and PLR. Interestingly, contrary to our findings, they observed that a higher mean body dose was associated with lower PLR (43). Our work supports the idea of reducing dosimetric parameters, particularly bone V10Gy, as a strategy to improve treatment outcomes in STS.

This research has several limitations. The small sample size and retrospective design may introduce selection bias and limit the generalizability of the findings. A major limitation is the lack of a validation cohort, which is necessary to confirm the robustness and generalizability of ΔPLR as prognostic marker and bone V10 Gy < 362 cc as a dosimetric parameter. Additionally, dosimetric studies of sarcomas present inherently challenges due to the diverse patient population, variations in sarcoma presentation, and heterogeneity in treatment options. Although we implemented strict inclusion criteria and utilized multivariate analyses to address these issues, the small sample size and the number of analyses conducted require caution in interpreting the results. Further research with larger cohorts is needed to validate these findings and assess their applicability to a broader sarcoma patients.

Current findings provide additional support for the significance of routinely collected hematologic markers, such as NLR and PLR, as important prognostic indicators in patients receiving therapy for STS. Furthermore, our results suggest that minimizing the rise in PLR following RT by keeping the bone V10Gy under 362 cc could potentially enhance beneficial outcomes in STS.

## Data Availability

The raw data supporting the conclusions of this article will be made available by the authors, without undue reservation.

## Supplementary material

Supplementary Table 2 Disease-free survival data by sarcoma histological subtype, stratified by ΔPLR and pre-RT/post-RT NLR cut-offs.
