# Supplementary material for: A study of pre- and post-treatment hematologic markers of immune response in patients undergoing radiotherapy for soft tissue sarcoma
Source: Front Oncol. 2024 Oct 3;14:1392705. doi: 10.3389/fonc.2024.1392705 (PMC11484061; doi:10.3389/fonc.2024.1392705)
Supplement: Supplementary file 1 [file Table1.docx]

| **Clinical Factor** | **Overall Survival, HR (95% CI)** | |  | **Disease-Free Survival, HR (95% CI)** | |
| --- | --- | --- | --- | --- | --- |
|  | **Univariate** | **Multivariate** |  | **Univariate** | **Multivariate** |
| Pre-RT ANC | 1.37 (1.02-1.82)* | NS |  | 1.33 (1.09-1.63)** | 1.21 (1.01-1.45)* |
| Pre-RT ALC | NS | - |  | NS | - |
| Pre-RT PLT | NS | - |  | NS | - |
| Pre-RT NLR | 1.27 (1.05-1.53)* | 1.25 (1.03-1.51)* |  | 1.29 (1.11-1.51)** | 1.31 (1.11-1.53)** |
| Pre-RT PLR | 1.79 (1.16-2.76)** | NS |  | 1.91 (1.35-2.71)** | 1.60 (1.10-2.32)* |
| 3 mo post-RT ANC | NS | - |  | NS | - |
| 3 mo post-RT ALC | 0.88 (0.79-0.99)* | NS |  | 0.85 (0.78-0.94)** | 0.89 (0.81-0.98)* |
| 3 mo post-RT PLT | 1.91 (1.16-3.15)* | 2.05 (1.26-3.31)** |  | 1.80 (1.20-2.68)** | 2.06 (1.42-3.08)** |
| 3 mo post-RT NLR | NS | - |  | 1.04 (1.01-1.08)* | NS |
| 3 mo post-RT PLR | 1.26 (1.10-1.44)** | 1.23 (1.05-1.44)** |  | 1.29 (1.15-1.45)** | 1.23 (1.08-1.40)** |
| 6 mo post-RT ANC | NS | - |  | NS | - |
| 6 mo post-RT ALC | 0.82 (0.72-0.94)** | 0.78 (0.66-0.91)** |  | 0.90 (0.83-0.97)** | 0.90 (0.82-0.98)* |
| 6 mo post-RT PLT | NS | - |  | NS | - |
| 6 mo post RT NLR | 1.11 (1.04-1.19)** | 1.18 (1.07-1.30)** |  | 1.06 (1.01-1.12)* | NS |
| 6 mo post-RT PLR | 1.56 (1.23-1.97)** | 1.86 (1.36-2.54)** |  | 1.41 (1.17-1.70)** | 1.47 (1.16-1.86)** |
| 12 mo post-RT ANC | NS | - |  | NS | - |
| 12 mo post-RT ALC | 0.73 (0.60-0.89)** | 0.69 (0.54-0.88)** |  | 0.91 (0.84-0.99)* | NS |
| 12 mo post-RT PLT | NS | - |  | NS | - |
| 12 mo post-RT NLR | 1.19 (1.06-1.34)** | 1.27 (1.07-1.51)** |  | NS | - |
| 12 mo post-RT PLR | 2.04 (1.25-3.33)** | 2.05 (1.20-3.52)** |  | 1.89 (1.28-2.78)** | 1.73 (1.16-2.56)** |

*p < 0.05

**p < 0.01

**Supplemental Table 1.** Cox proportional hazards regression analysis of clinical factors and hematologic markers and association with overall survival and disease-free survival. Multivariate model controlled for surgical resection and receipt of chemotherapy.
